# A case of osteoporotic fracture leading to diagnosis of Klinefelter syndrome in a 76-year-old man

**DOI:** 10.1210/jcemcr/luag035

**Published:** 2026-03-13

**Authors:** Joshua Nathan Shapiro, Mihaela Oprea

**Affiliations:** Division of Endocrinology, Diabetes & Metabolism, State University of New York, Downstate Medical Center, Brooklyn, NY 11203, USA; Division of Endocrinology, Diabetes & Metabolism, Veterans Affairs New York Harbor Healthcare System, Brooklyn, NY 11209, USA; Division of Endocrinology, Diabetes & Metabolism, Veterans Affairs New York Harbor Healthcare System, Brooklyn, NY 11209, USA

**Keywords:** Klinefelter syndrome, primary hypogonadism, osteoporosis, male infertility, sex chromosome aneuploidy

## Abstract

Klinefelter syndrome (KS) is the most prevalent sex chromosome disorder in men but often remains clinically silent until later life. We describe an elderly man whose evaluation for a rib fracture uncovered the diagnosis. A 76-year-old male presented after a low-impact fracture during a coughing episode. Clinical history revealed delayed sexual maturation, small testes, infertility, and mild gynecomastia. Hormonal testing demonstrated markedly reduced testosterone with elevated luteinizing and follicle-stimulating hormones, suggesting primary testicular failure. Karyotype analysis confirmed 47,XXY. Bone mineral density testing revealed osteopenia, meeting criteria for clinical osteoporosis. He received intravenous zoledronic acid, while androgen therapy was postponed pending urologic assessment. This case emphasizes that KS can remain undetected for decades and highlights the need to consider hypogonadism in men with unexplained bone loss. Early recognition facilitates appropriate treatment and long-term monitoring for associated systemic complications.

## Introduction

Klinefelter syndrome (KS), characterized by the 47,XXY karyotype, is the most common sex chromosome aneuploidy in males. Despite an estimated prevalence of 1 in 600 in the general population, nearly two-thirds of patients remain undiagnosed through adulthood [1]. Hypogonadism associated with KS may contribute to metabolic syndrome and Type 2 diabetes, infertility, and skeletal fragility [2]. Osteoporosis or low-trauma fracture in men warrants evaluation for secondary causes including screening for low testosterone [3]. We report a case of a 76-year-old man whose fragility rib fracture led to the diagnosis of KS in late adulthood.

## Case presentation

A 76-year-old man was referred to the endocrinology clinic after sustaining a right ninth rib fracture during a severe coughing episode associated with an upper respiratory infection. The low-trauma nature of the fracture prompted evaluation for osteoporosis and secondary causes of bone loss.

He reported delayed puberty and lifelong small testicular size, but denied cryptorchidism, hypospadias, or micropenis. He noted that when he was around 2 years old his pediatrician pointed out that he had small testes. Later during his adolescence, he was teased for appearing underdeveloped, specifically for having smaller testicles and sparse body hair. These findings were also relayed to him by his family physician, but no further investigation took place. Mild gynecomastia developed later in life. His fertility history was notable for multiple miscarriages with his first wife and a semen analysis in his 30s showing low sperm count, however he reports the miscarriages were attributed to his wife's type 1 diabetes. Although further evaluation was offered at that time, he declined to undergo further workup because he thought the etiology was due to his wife's type 1 diabetes. Approximately 10-15 years prior to his first endocrine encounter, a urologist informed him of low testosterone levels, but he declined treatment. For many years, he experienced erectile dysfunction and decreased libido.

His medical history was significant for hypertension, coronary artery disease, atrial fibrillation, benign prostatic hyperplasia, hypothyroidism, chronic kidney disease (stage IIIa), asthma, calcium oxalate nephrolithiasis, gastroesophageal reflux disease with Barrett esophagus, lower gastrointestinal bleeding, and cervical spine radiculopathy.

Family history was significant for a great-nephew who had congenital anomalies, though he was unable to elaborate further on the genetics of the condition. His 2 brothers were healthy, and both fathered children. No known family history of infertility, neuromuscular disorders, or genetic syndromes.

### Diagnostic assessment

Physical examination revealed a height of 185.4 cm (height standard deviations ≈ + 1.2 based on CDC adult male reference data) [4], weight of 93 kg, and mild bilateral gynecomastia and central adiposity. Genital examination by his urologist revealed bilaterally descended but small nontender testicles without masses. The patient refused further physical examination of his testicles in our clinic, including the use of an orchidometer. Laboratory testing revealed low testosterone with elevated gonadotropins, consistent with primary hypogonadism without evidence of anemia (Table 1). Due to the patient's lifelong history of microorchidism, infertility, oligospermia, delayed puberty, low testosterone with elevated gonadotropins, erectile dysfunction, decreased libido, as well as unexplained low bone mineral density (BMD), a chromosomal karyotyping was performed, which showed a 47,XXY karyotype, confirming KS.

**Table 1:** Laboratory evaluation in our patient

Test	Result	Normal Range
Total testosterone by mass spectrometry	**159.2 ng/dL (5.53 nmol/L)**	264-916 ng/dL (9.2-31.8 nmol/L)
Free testosterone by mass spectrometry	**3.60 ng/dL (0.12 nmol/L)**	5.0-21.0 ng/dL (0.17-0.73 nmol/L)
% Free testosterone	2.26%	1.50-4.20%
LH	**27.63 mIU/mL (27.63 IU/L)**	1.24-8.62 mIU/mL for male (1.24-8.62 IU/L)
FSH	**62.60 mIU/mL (62.60 IU/L)**	1.27-19.26 mIU/mL for male (1.27-19.26 IU/L)
SHBG	40.2 nmol/L (40.2 nmol/L)	19.3-76.4 nmol/L (19.3-76.4 nmol/L)
Ionized calcium	5.1 mg/dL (1.27 mmol/L)	4.5-5.6 mg/dL (1.12-1.40 mmol/L)
PTH	55.1 pg/mL (5.8 pmol/L)	18.5-88.0 pg/mL (1.96-9.33 pmol/L)
C-telopeptide	346 pg/mL (346 ng/L)	38-724 pg/mL (38-724 ng/L)
Propeptide type I collagen	60.1 ng/mL (60.1 µg/L)	18.3-86.1 ng/mL (18.3-86.1 µg/L)
Osteocalcin	17.6 ng/mL (17.6 µg/L)	4.1-25.0 ng/mL (4.1-25.0 µg/L)
25OH vitamin D	46.69 ng/mL (116.7 nmol/L)	15-90 ng/mL (37.5-225 nmol/L)
Na	141 mmol/L (141 mmol/L)	135-145 mmol/L (135-145 mmol/L)
Hgb	13.6 g/dL (136 g/L)	13-18 g/dL (130-180 g/L)

Abnormal values are shown in bold font. Values in parenthesis are reported in the International System of Units (SI).

Abbreviations: 25OH vitamin D, (25-hydroxyvitamin D); FSH, follicle-stimulating hormone; Hgb, Hemoglobin; LH, Luteinizing Hormone; Na, Sodium; PTH Parathyroid Hormone; SHBG, Sex Hormone-Binding Globulin.

Chest computed tomography was notable for a nondisplaced right lateral ninth rib fracture. Dual-Energy X-ray Absorptiometry scan demonstrated lumbar spine T-score −2.0 with BMD 0.83 g/cm^2^ (SI: 8.3 kg/m^2^), left total hip T-score −2.0 with BMD 0.697 g/cm^2^ (SI: 6.97 kg/m^2^), and femoral neck T-score −2.1 with BMD 0.613 g/cm^2^ (SI: 6.13 kg/m^2^).

Fracture Risk Assessment Tool estimated a 10-year hip fracture risk of 4.9% and a 10-year major osteoporotic fracture risk of 14%.

## Treatment

Testosterone therapy was deferred pending urologic evaluation for benign prostatic hyperplasia. Given his fragility fracture, consistent with clinical osteoporosis, treatment was initiated with intravenous zoledronic acid. Additionally, he was advised to maintain adequate calcium and vitamin D intake. Genetic counseling for KS was provided, and the patient was educated about ongoing recommended screening for associated disorders, ie, screening mammogram.

## Outcome and follow-up

Follow-up bone density examination showed improvement of BMD following infusion of zoledronic acid. The patient continues to be followed up in endocrine clinic and is appreciative of the investigation into his secondary cause of osteoporosis. He continues to be followed with urology for possible initiation of testosterone therapy.

## Discussion

This case highlights a delayed diagnosis of KS in late adulthood. Klinefelter syndrome often goes unrecognized due to variable clinical presentation. Our patient exhibited hallmark features of KS, including small testes, oligospermia, primary hypogonadism, infertility, and mild gynecomastia. Hypogonadism contributes to reduced BMD through decreased bone formation and increased bone resorption. In addition to the effect of reduced testosterone, decreased Leydig cell secretion of insulin-like factor 3 leads to impaired osteoblast function, predisposing to osteoporosis [5]. Up to 48% of men with KS have osteopenia and 6% to 15% have osteoporosis [5].

While osteoporosis screening is routinely recommended in men aged 70 years and older due to age-related bone loss [6], this patient's fracture occurred in the setting of minimal trauma, raising concern for underlying skeletal fragility. We acknowledge that rib fractures are variably classified as fragility fractures and that age-related decline in BMD likely contributed to his presentation. However, longstanding untreated primary hypogonadism associated with KS likely impaired peak bone mass acquisition and contributed to chronic bone loss, representing an important risk factor.

KS is also associated with increased risk of metabolic syndrome, venous thromboembolism, and breast cancer. European guidelines for adults with KS recommend long-term surveillance for metabolic, cardiovascular, and endocrine complications. They specifically advise thrombosis prophylaxis prior to long flights, as well as routine clinical breast and axilla exam, along with mammography, to screen for breast cancer given the increased risk associated with the presence of 2 X chromosomes [7]. While testosterone therapy may improve bone density and body composition, antiresorptive therapy remains appropriate in patients with high fracture risk. Early recognition of KS provides an opportunity for targeted treatment and risk reduction.

## Learning points

- Klinefelter syndrome can remain undiagnosed well into late adulthood, particularly in patients with subtle symptoms.
- In elderly men presenting with osteoporotic fractures and primary hypogonadism, karyotype analysis should be considered to uncover potential chromosomal abnormalities.
- Timely diagnosis allows for targeted management including testosterone replacement therapy and osteoporosis treatment, which may improve quality of life and long-term outcomes.

## Contributors

All authors made individual contributions to authorship. J.N.S. and M.O. were involved in the diagnosis and management of the patient and manuscript submission.

## Data Availability

Original data generated and analyzed during this study are included in this published article.
