# Correction to: “A case of osteoporotic fracture leading to diagnosis of Klinefelter syndrome in a 76-year-old man”

**DOI:** 10.1210/jcemcr/luag104

**Published:** 2026-03-27

In the above-named article by Shapiro JN and Oprea M (*JCEM Case Reports*. 2026, 4(3); doi: 10.1210/jcemcr/luag035), there was an error in the Case presentation section.

In the originally published article, in the Case presentation section, in the second paragraph, the sixth sentence was originally published as, “His fertility history was notable for multiple miscarriages with his first wife and a semen analysis in his 30 seconds showing low sperm count, however he reports the miscarriages were attributed to his wife’s type 1 diabetes.” In the corrected article, “30 seconds” has been updated to “30s” and the sentence now reads, “His fertility history was notable for multiple miscarriages with his first wife and a semen analysis in his 30s showing low sperm count, however he reports the miscarriages were attributed to his wife’s type 1 diabetes.”

The publisher regrets this error.

The article has been corrected online.

doi: 10.1210/jcemcr/luag035

